# Prediction of Lap Shear Strength and Impact Peel Strength of Epoxy Adhesive by Machine Learning Approach

**DOI:** 10.3390/nano11040872

**Published:** 2021-03-30

**Authors:** Haisu Kang, Ji Hee Lee, Youngson Choe, Seung Geol Lee

**Affiliations:** 1 School of Chemical Engineering, Pusan National University, Busan 46241, Korea; haisu@pusan.ac.kr (H.K.); jihee9816@pusan.ac.kr (J.H.L.); 2 Department of Chemical and Biomolecular Engineering, Pusan National University, Busan 46241, Korea; 3 Department of Organic Material Science and Engineering, Pusan National University, Busan 46241, Korea

**Keywords:** epoxy adhesive, machine learning, artificial neural network, lap shear strength, impact peel strength

## Abstract

In this study, an artificial neural network (ANN), which is a machine learning (ML) method, is used to predict the adhesion strength of structural epoxy adhesives. The data sets were obtained by testing the lap shear strength at room temperature and the impact peel strength at −40 °C for specimens of various epoxy adhesive formulations. The linear correlation analysis showed that the content of the catalyst, flexibilizer, and the curing agent in the epoxy formulation exhibited the highest correlation with the lap shear strength. Using the analyzed data sets, we constructed an ANN model and optimized it with the selection set and training set divided from the data sets. The obtained root mean square error (RMSE) and *R*^2^ values confirmed that each model was a suitable predictive model. The change of the lap shear strength and impact peel strength was predicted according to the change in the content of components shown to have a high linear correlation with the lap shear strength and the impact peel strength. Consequently, the contents of the formulation components that resulted in the optimum adhesive strength of epoxy were obtained by our prediction model.

## 1. Introduction

Structural adhesives are used in structural joints of transport devices such as automobiles or aircraft. Compared with bonding techniques such as conventional screws, mechanical coupling with bolts, or welding, structural adhesives deliver outstanding performance in increasing the structural stiffness, reducing vibrational noise, and reducing the weight of structures. In the early stage of their development, the use of structural adhesives was limited to the structural junctions of transport vehicles. However, structural adhesives are now broadly used for high-performance and customized structural designs, and the range of joints on which structural adhesives are used has been extended, which has greatly increased the demand for structural adhesives [1,2,3].

To be used for structural bonding, a structural adhesive is required to exhibit excellent adhesion strength in addition to superb resistance to impact and flexural deformation. An epoxy adhesive is a representative structural adhesive and is the most widely used adhesive with qualified performance. Epoxy adhesives largely consist of two parts: an epoxy resin and a curing agent. Epoxy resin is a thermosetting resin with various functional groups in its chemical structure, including epoxy (–O–) groups, which react with amino (–NH) groups in the curing agents. The reaction between epoxy groups in an epoxy resin and amino groups in a curing agent, i.e., the curing reaction, results in a crosslinking structure, which improves heat resistance, solvent resistance, and moisture resistance [2,3]. In practical applications, the epoxy resin is often modified for a specific purpose, or its additive composition is varied for desired properties. For this purpose, epoxy adhesives require highly advanced formulation techniques when applied in transport vehicles as structural adhesives, where they are combined with various additives. Numerous additives have been extensively studied so far, including catalysts, flexibilizers, tougheners, fillers, reactive diluents, and promoters [4,5,6,7,8,9,10,11,12]. The catalysts are mainly imidazoles containing methyl (–CH_3_) functional groups; these catalysts open the epoxy ring and improve the curing reaction [4]. A flexibilizer and tougheners are added to improve the mechanical properties of epoxy adhesives. Urethane-based polymers are widely used as flexibilizers, which enhance the mechanical properties of epoxy adhesives through phase separation with the epoxy resin [5,6]. The effect of tougheners consisting of core–shell rubber (CSR) particles has recently been studied, and the results suggest that the addition of such tougheners improves the peel strength and impact resistance of the adhesive [7,8]. Reactive diluents are used to reduce the viscosity of epoxy adhesives, and promoters smooth the joint surface, efficiently enhancing the adhesion strength [4]. 

In addition to variations in the types of additives, formulations can be diversified according to their content, curing conditions, and application temperature, which greatly influence the properties of adhesives. However, the large number of possible formulations and their complexity make quantitative predictions of adhesive properties, or even predictions of their basic properties, challenging. A systematic method for optimizing formulations is needed. As one approach to this problem, machine learning (ML) represents a powerful potential solution. 

ML is a data analysis method that provides computers the ability to learn without being explicitly programmed. Unlike conventional statistical data analysis, in which individual analytical models are developed on the basis of each data point, ML learns new data on its own with a model developed by accumulating training experiences from a given data set [13]. Commonly used ML methods include generalized linear regression [14], decision trees [15], random forest [16], support vector machine [17], and artificial neural networks (ANNs) [18]. In particular, ANNs, which are also known as deep learning if the neural network has multiple deep layers, provide the most accurate predictions in a short time from highly disordered data through their high-level algorithms [18]. In recent reports, the use of ANNs in materials science has been successfully demonstrated [19,20,21]. For instance, the tensile shear strength of bonded beechwood was predicted by an ANN model trained using an experimental database [22]. In addition to ANNs being trained with experimental databases, they have also been trained from data sets calculated through density functional theory in applications where they were used to predict redox potentials on the basis of the structure of anode materials for lithium-ion batteries [20] or to screen possible perovskite structures [19].

Therefore, in the present study, an ANN was used as an ML method to predict basic adhesion strength on the basis of the formulation of high-performance epoxy adhesives. Various epoxy adhesive formulations with different contents of basic components were prepared as high-strength structural adhesives. The lap shear strength and impact peel strength of the adhesive with each formulation were obtained experimentally at 25 °C and −40 °C, respectively, to build a database of adhesion strength. The experimental database was first analyzed statistically, and the correlation between variables was examined. The ANN model was built and trained using the examined database and optimized to have high predictability. The optimized ANN model was verified by the linear regression method and errors. Consequently, the adhesion strength was predicted from the content of the critical component, which has been a challenge in this field. Thus, we believe that the approach implemented in the present study will be a cornerstone toward predicting complex formulations of adhesives with targeted properties. 

## 2. Materials and Methods

### 2.1. Materials for Formulation 

Diglycidyl ether bisphenol-A (EEW: 184−190, Kukdo Chemical) as an epoxy resin, dicyandiamide (DICY, Sigma-Aldrich) as a curing agent, 1-cyanoethyl-2-4-metylimidazole (2E4MZ-CNS, Sigma-Aldrich) as a catalyst, and an amino silane-based coupling agent (promoter) were used as received. Hydroxyl-terminated polyurethane (HTPU, Huntsman) and CSR (core–shell rubber) particles (methacrylate-butadiene-styrene (MBS), Rohm & Haas, diameter: 150 nm) were used as a flexibilizer to improve the flexibility and toughness of epoxy blends. As reactive diluent, 1,6-hexanediol diglycidyl ether (HDDGE)), was used. Fumed silica with ~20 nm in diameter as a filler was used to control the thixotropic property of the adhesive.

### 2.2. Mechanical Properties Test Procedure

For the preparation of the formulated epoxy system, the epoxy resin, modifiers (HTPU and CSR), and diluent were blended in a high-speed vacuum mixer (ARV-310, Thinky, Tokyo, Japan) at 60 ℃ for 30 min and then particles, including curing agents, silica, and catalyst, were poured onto the prepared blend and mixed together under vacuum condition by high-speed vacuum mixer (ARV-310, Thinky, Tokyo, Japan) at room temperature for 30 min. The viscosity of formulated adhesive was about 1,200,000 cps at 21 ℃. This paste-type adhesive mainly contained liquid epoxy resin, modifiers, diluent, curing agent, and nano-sized fumed silica.

**Figure 1:**
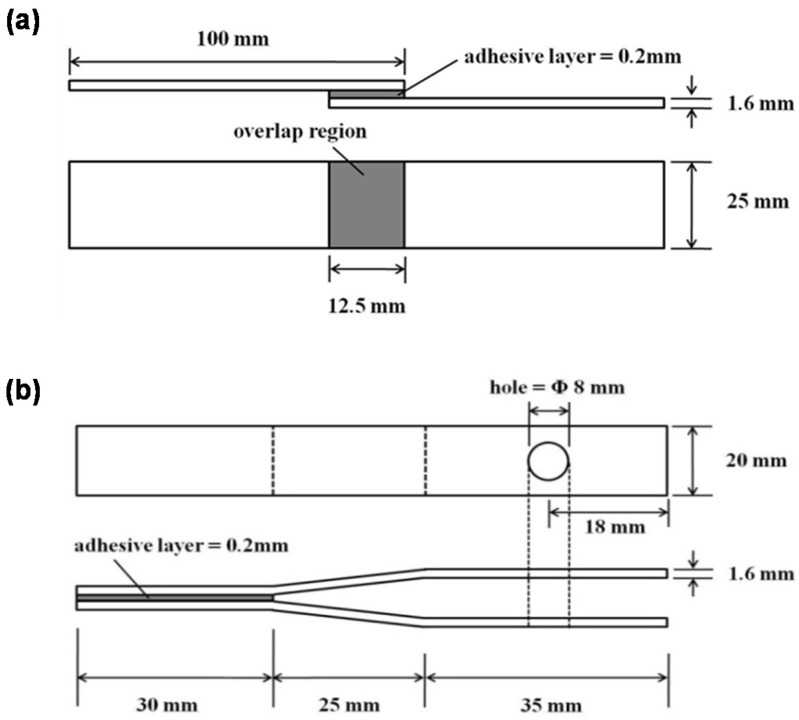
The specimens for (**a**) the lap shear strength test (ASTM D 1002) and (**b**) the impact peel strength test (ISO 11343).

Specimens were prepared to measure the mechanical properties of epoxy blends. The adhesive was applied by using a knife spreader onto a substrate. The thickness of the adhesive layer was precisely controlled by a glass bead, as a spacer, the size of which was 180~200 µm in diameter. Specimens were cured at 180 ℃ for 30 min in a convection oven with a one-step process. Two clamps were used to hold the specimen in the right position during the curing process. After the curing process, all samples were placed at room temperature for 24 h for aging. The material of all specimens was SPRC440, which is a high-strength steel plate. The lap shear strength and impact peel strength of the specimens were measured based on ASTM D 1002 and ISO 11343s, as shown in Figure 1a,b, respectively. For the impact peel test, two symmetry holes were needed for hookup onto the right position of the test instrument. For the perfect alignment between the specimen and holding zig, the center point in the vertical direction was marked on both specimen and holding zig.

Lap shear strength was measured by TINUS OLSEN 25ST instrument (Horsham, PA, USA) at 21 ℃, and impact peel strength by INSTRON CEAST 9350 instrument (Norwood, MA, USA) at −40 °C. The impact peel specimen was placed in the bottom chamber of the instrument, in which the extremely low temperature was controlled automatically by liquid nitrogen. The failure mode of all specimens for lap shear and impact peel tests was cohesive, which could be achieved by the precise tuning of each adhesive formulation. The dimensional tolerance of specimens existed in the overlap region of adhesive-applied area, which was 12.5~13 mm in the length direction. For the lap shear test, two specimens were used, and for the impact peel test, three specimens were used. 

The input data set for the artificial neural network (ANN) model is presented in Table 1.

### 2.3. Machine Learning Procedure

In this study, among various ML techniques, ANN was selected to build a prediction model from given experimental data sets for lap shear strength at room temperature and impact peel strength at −40 °C because ANN is the most effective technique for classifying and predicting complex nonlinear or linear relationships among numerous variables [20,21,23,24]. In the input data set for the ANN model, lap shear strength, and impact peel strength were set as target variables, and two separate ANN models were constructed to have only one target variable. For each individual ANN model, we set a total of eight components (i.e., resin, CSR, flexibilizer, diluent, filler, promoter, curing agent, catalyst) as input variables. Each component was fixed to one type in order to efficiently optimize the ANN model by a small number of input variables [25]. The values of each variable in the data set were prepared by weight; thus, an adequate number of data samples were prepared by simply changing the weight ratio of each component. Each data sample was based on the practical performance, the reference values of which were ~35 MPa for lap shear strength and ~40 N/ mm for impact peel strength [26,27,28]. All the values of each variable were scaled by the minimum and maximum values as standard (min–max normalization). Twenty percent of the total data samples were used as a selection set to set initial hyperparameters of the ANN model (i.e., the number of hidden layers and neurons), and 60% of the total data samples except the selection set were classified as a training set with which to train the initial ANN model; the rest of the samples were classified as a test set used to analyze and verify the trained ANN model. Selection sets were chosen as the most consistent samples without missing values, whereas the training set and test set were divided randomly. When the ANN model was trained, weight and bias were randomly set as initial values, and the activation function for adjusting weight and bias was used as a hyperbolic tangent function (TanH) in the sigmoid function series. The ANN model was trained by the quasi-Newton method, which is an optimization method widely used in a wide range of numerical applications. Whereas the Newton method requires calculating the Hessian matrix, consisting of the second derivative of the loss function at each iteration, the quasi-Newton method enables efficient calculation in a short time by predicting the inverse Hessian matrix at each iteration with only the first partial derivatives of a loss function. Various ANN models have been optimized by the quasi-Newton method and have been reported to have high predictability [24]. Thus, our ANN models were trained by the quasi-Newton method with the normalized squared error (NMSE) set as a loss function with a maximum of 5000 iterations to optimize the weight and bias parameter in our models. All processes were performed with the Neural Designer software [29].

## 3. Results and Discussion

### 3.1. Data Analysis

**Figure 2:**
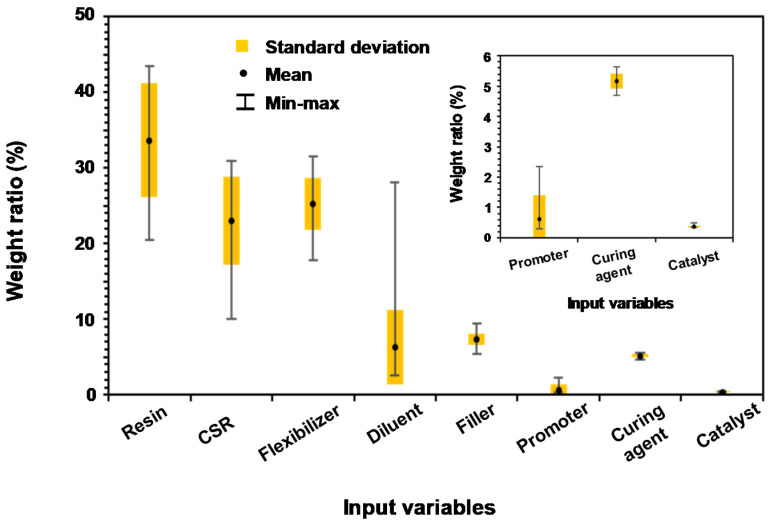
Statistical analysis of the minimum, maximum, and average values (with standard deviation) of the input variables in the data sets.

Prior to the construction of the ANN model, the data set obtained from experiments (Table 1) was analyzed. In order to verify the distribution of the data sets for accurate optimization of the ANN model, the average, minimum, maximum, and standard deviation of the values for each variable, which was analyzed by the weight ratio (%), are shown in Figure 2. In Figure 2, resin, CSR, flexibilizer, and diluent composed most of the mass in the formulation and had a wide range of values compared with the other variables. Specifically, the diluent only exhibited an average weight ratio of 6.3, whereas the maximum value was 28, which is regarded as an exceptional case. By contrast, the filler, promoter, curing agent, and catalyst were limited to a small proportion of the formulation; the weight ratio of the catalyst, in particular, ranged from only 0.35 to 0.5. 

**Figure 3:**
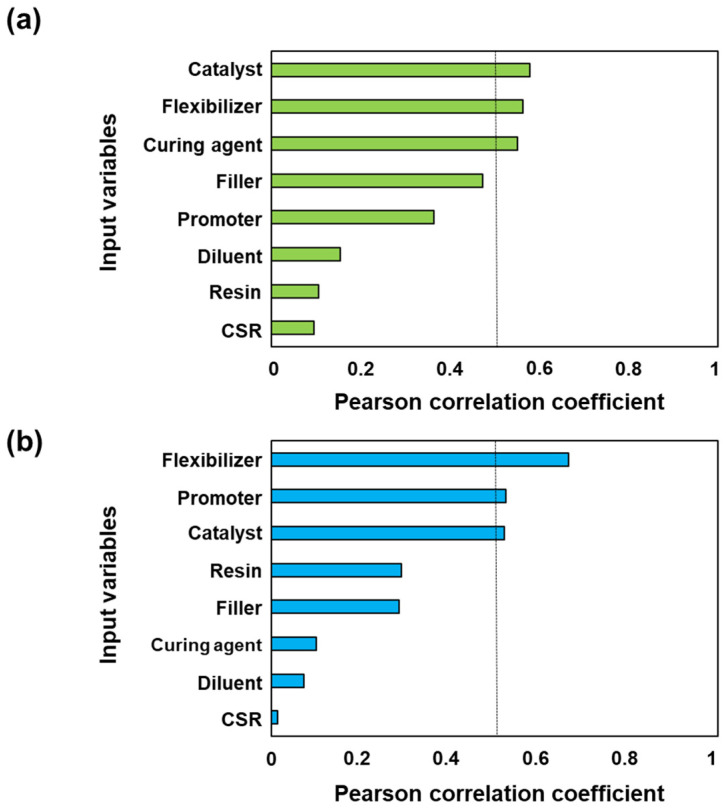
Pearson correlation coefficients (**a**) between input variables and lap shear strength and (**b**) between input variables and impact peel strength.

We performed a linear correlation analysis between the input and target variables in the data set to understand the extent to which the values in each variable affected the lap shear strength and impact peel strength; the results are presented in Figure 3. Linear correlation analysis was carried out by calculating the Pearson correlation coefficient (PCC), for which an absolute value of 1 indicates a perfect linear correlation and a value of 0 indicates the absence of linear correlation. Figure 3a indicates the PCC between input variables and lap shear strength. The PCC for the catalyst, flexibilizer, and curing agent were all greater than 0.5; the catalyst, in particular, was found to exhibit the strongest correlation with the lap shear strength. This result is reasonable because the catalyst stimulates rapid ionic polymerization of the epoxy compound, whose content needs to be precisely controlled. However, in Figure 3b, which shows the PCCs between the input variables and the impact peel strength, the flexibilizer, promoter, and catalyst show PCCs greater than 0.5; however, the flexibilizer, in particular, was found to be the variable most strongly correlated with the lap shear strength. The flexibilizer exhibited the highest PCC, which is reasonable because the flexibilizer plays key role in enhancing toughness, which is reflected in the impact peel strength at −40 °C. Interestingly, the catalyst, curing agent, and promoter in Figure 3a,b show a relatively high PCCs despite their small proportion of the total weight of the adhesive. By contrast, the correlations of the diluent, resin, and CSR (which dominated the total weight of the adhesive) with the lap shear strength and impact peel strength resulted in PCCs less than 0.2. Thus, critical components strongly impact the lap shear strength and the impact peel strength, even if they represent only a small portion of the total weight. 

Moreover, given that the average lap shear strength and impact peel strength in the data set were 36.68 MPa and 39.08 N/ mm, respectively, all data samples satisfied the general requirements to be used as a structural adhesive (lap shear strength > 28 MPa, impact peel strength > 20 N/mm). Therefore, the critical components with high correlation with the lap shear strength and impact peel strength are considered to effect further enhancement of the adhesive strength. 

### 3.2. Training Artificial Neural Network

**Figure 4:**
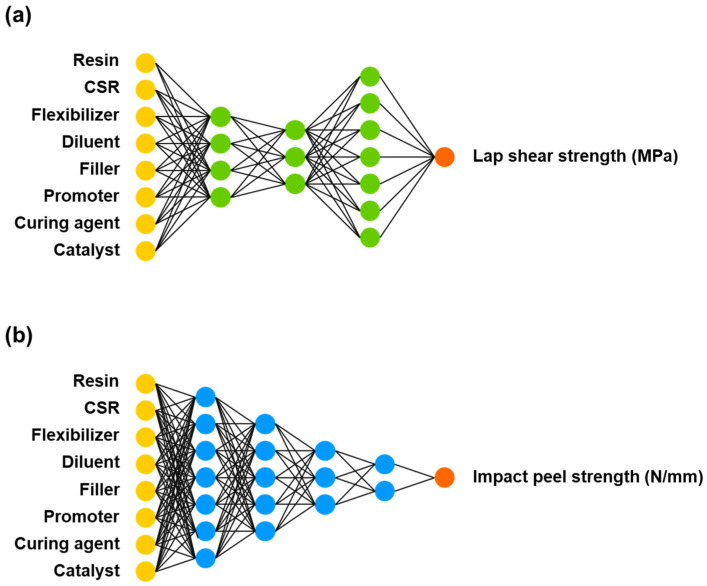
Artificial neural network (ANN) models for predicting (**a**) lap shear strength and (**b**) impact peel strength.

An ANN model was initially constructed by training a selection set of the data set with the hyperparameters to achieve the lowest error. The hyperparameters in the ANN were the number of hidden layers and neurons, which were adjusted to three hidden layers and four, three, and seven neurons for each layer for the lap shear strength prediction model (Figure 4a). For the impact peel strength prediction model, four hidden layers and seven, five, three, and two neurons for each layer were adjusted (Figure 4b). The initial models were then trained with a training set and optimized for minimum loss. The optimized ANN model was used to predict the lap shear strength and impact peel strength with the training set and the test set; the calculated root mean square error (RMSE) and normalized squared error (NSE) for each prediction are presented in Table 2. 

In the lap shear strength prediction model, the training set and test set showed low RMSEs of 0.053 and 0.590, respectively. Given that the standard deviation of the lap shear strength values was 1.019 in the data set, our ANN model for lap shear strength prediction showed excellent accuracy. For the impact peel strength prediction, our ANN model showed an RMSE of 1.730 in the training set and 8.218 in the test set. Given that the standard deviation of impact peel strength values was 5.590 in the data set, our ANN model for impact peel strength prediction showed good accuracy in the training set, whereas the test set prediction was slightly unstable.

**Figure 5:**
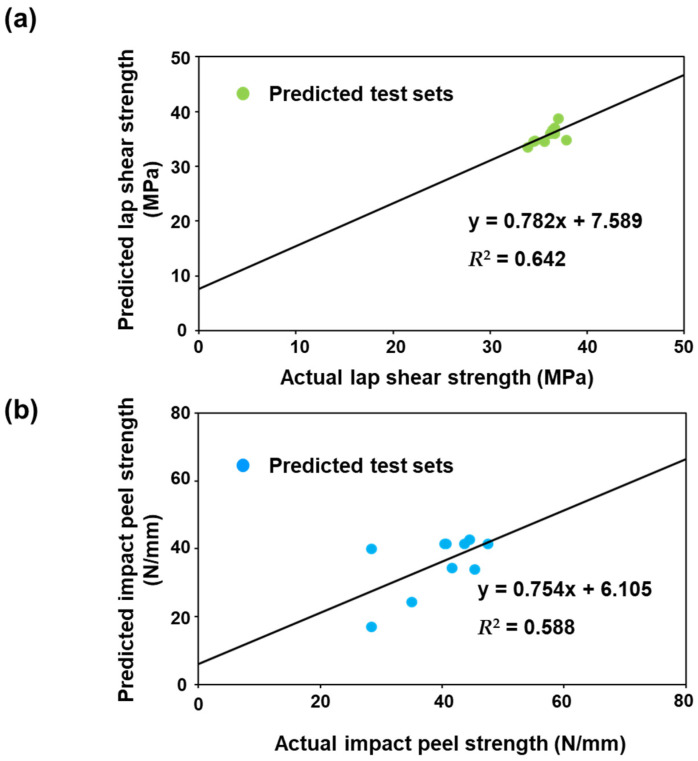
(**a**) Correlation analysis of the predicted lap shear strength by ANN with the actual lap shear strength and the (**b**) correlation of the predicted impact peel strength by ANN with the actual impact peel strength.

To verify our ANN model, we also conducted a linear regression analysis; the results are presented in Figure 5. The linear regression analysis was performed by searching the regression line between the predicted values and actual values in the data set. Figure 5 demonstrates that the lap shear strength and the impact peel strength predictions of our ANN model showed a linear correlation with actual values. The *R*-squared (*R*^2^) values, which are the coefficients of determination, were 0.642 and 0.588 for the lap shear strength and the impact peel strength prediction model, respectively, indicating reasonable accuracy of the ANN model. 

### 3.3. Prediction of Optimized Formulation

**Figure 6:**
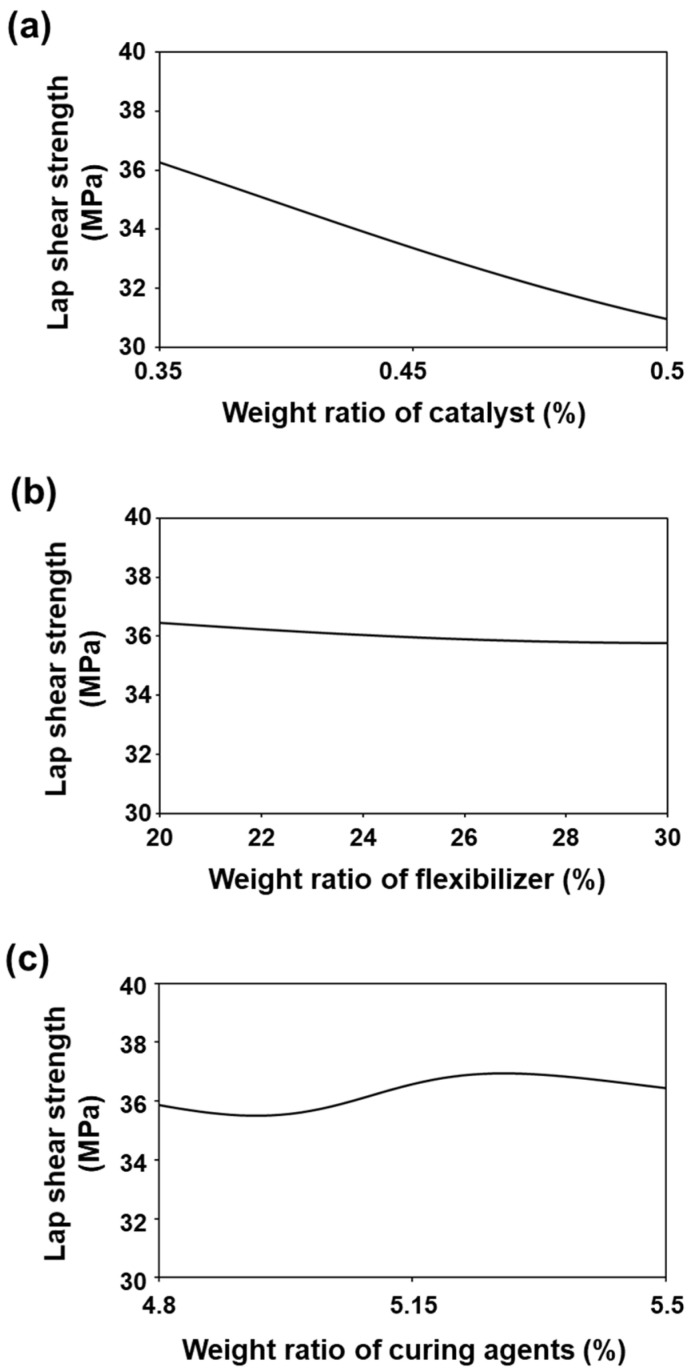
Prediction of the lap shear strength by the change in the weight ratio of the (**a**) catalyst, (**b**) flexibilizer, and (**c**) curing agents.

**Figure 7:**
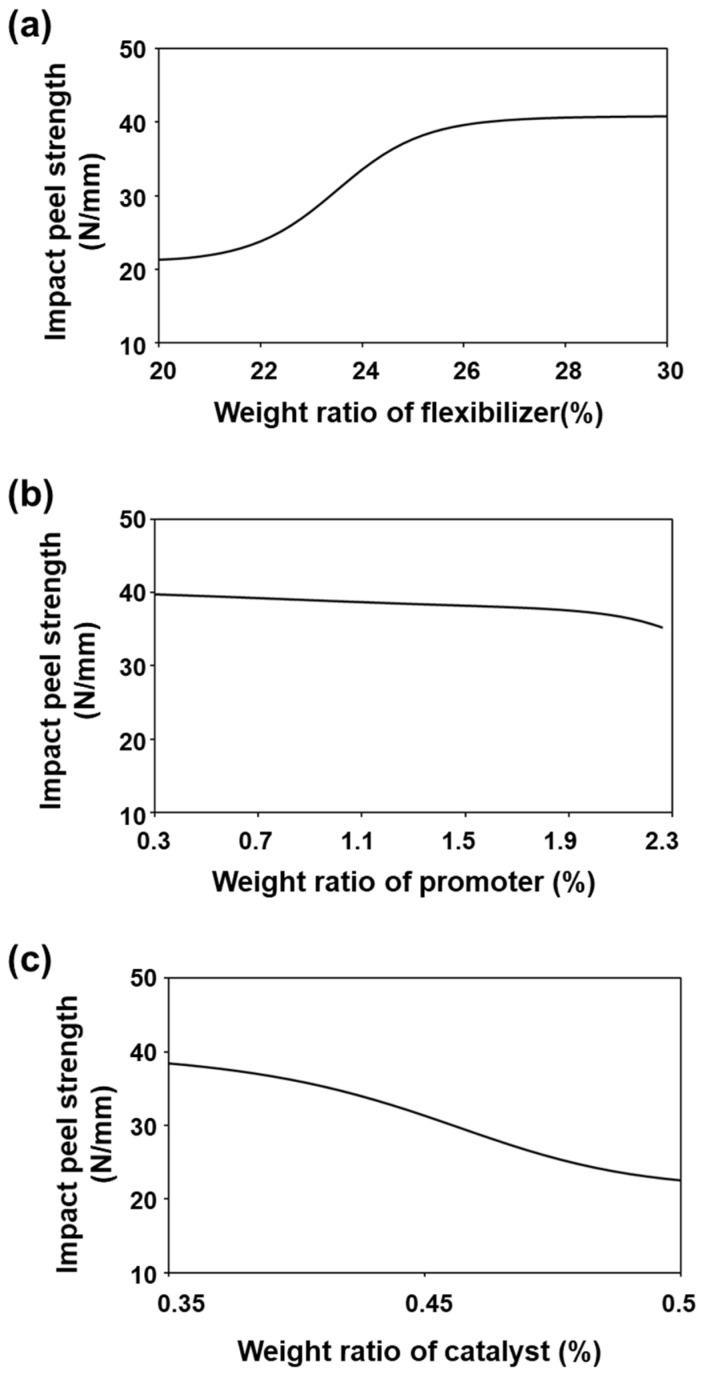
Prediction of impact peel strength by the change in the weight ratio of the (**a**) flexibilizer, (**b**) promoter, and (**c**) catalyst.

The effects of the input components on the lap shear strength and impact peel strength were examined by our ANN models (Figure 6 and Figure 7). Notably, among the input components, we considered only the critical components (i.e., those that showed a high linear correlation greater than 0.5 in Section 3.1).

The catalyst, flexibilizer, and curing agent were the input variables most strongly correlated with the lap shear strength; thus, the lap shear strength predicted by the change in weight ratio for each component is shown in Figure 6. Here, the other components were set to be constant at their average values. In Figure 6a, the lap shear strength is predicted to decrease when the weight ratio of catalyst changes from 0.35 to 0.5. This result is consistent with experimental studies showing that excess catalyst interrupts the improvement of adhesion strength because it leads to an inhomogeneous dispersion of catalyst [30]. As shown in Figure 6b, the lap shear strength was ~36 MPa when the weight ratio of flexibilizer was changed from 20 to 30, which indicates the lap shear strength was not significantly affected by the flexibilizer content. In Figure 6c, when the weight ratio of the curing agent was changed from 4.8 to 5.5, lap shear strength perturbed smoothly but showed relatively constant lap shear strength. Because the curing mechanism depends on the weight ratio of the curing agents, developing a crosslinking structure is critical to controlling the adhesion strength. However, understanding the curing mechanism has proven difficult even when a small number of components are used; the curing mechanism thus requires further study [31,32].

Flexibilizer, promoter, and catalyst were the input variables most strongly correlated with the impact peel strength; thus, the impact peel strength predicted by the change in weight ratio for each of these components is shown in Figure 7. As shown in Figure 7a, the impact peel strength saturated at its highest values when the weight ratio of the flexibilizer was greater than 25. This result indicates that a sufficient weight of flexibilizer can enhance the adhesion strength by inducing phase separation with the epoxy resin, especially under low-temperature conditions [33]. On the contrary, the impact peel strength did not substantially change with the change in the weight ratio of the promoter. Accordingly, we found that the promoter affected the impact peel strength upon its addition; however, the impact peel strength was not strongly affected by a change in content of the promoter [34]. Finally, the impact peel strength also rapidly decreased when the catalyst weight ratio was changed from 0.35 to 0.5 for the same reason that the lap shear strength decreased, as shown in Figure 7c. Consequently, we found that our ANN prediction model could reasonably predict the relationship between the formulation components and lap shear strength and impact peel strength, which enabled us to predict the optimum contents of the highly related components.

## 4. Conclusions

In this study, a prediction model for lap shear strength and impact peel strength according to the various formulations was developed by an ANN using an ML approach. First, we collected experimental adhesion data (lap shear strength and impact peel strength) for the various formulation samples. Each data sample included eight components: resin, CSR, flexibilizer, diluent, filler, promoter, curing agent, and catalyst. In the data analysis, we found that, among the formulation components, the catalyst, flexibilizer, and curing agent showed a high correlation with the lap shear strength. However, the flexibilizer, promoter, and catalyst showed a high correlation with the impact peel strength. The analyzed data were divided into a selection set, a training set, and a test set and then used to build and optimize the ANN models for predicting lap shear strength and impact peel strength. The optimized ANN model for lap shear strength exhibited RMSEs of 0.053 and 0.590, and that for impact peel strength exhibited RMSEs of 1.730 and 8.218 for the prediction of the training set and test set, respectively. Moreover, the optimized ANN model was examined by linear regression analysis, and the model showed *R*^2^ values of 0.642 and 0.588 for predictions of the lap shear strength and impact peel strength, respectively. On the basis of the analyses from our optimized ANN models, we regarded our ANN models to be adequate for predicting lap shear strength and impact peel strength with fairly reasonable predictability. Accordingly, we analyzed the predicted relationships between adhesion strength and the various components, which revealed the highest PCC and provided directional information about the optimum content of each component for high lap shear strength and impact peel strength. Thus, as a cornerstone toward predicting complex formulations of adhesives with specific target properties, we believe that our study provides a new path for developing high-strength structural adhesives.

## Figures and Tables

**Table 1:** Data set from adhesion test for machine learning.

Sample	Resin (g)	Core Shell Rubber (g)	Flexibilizer (g)	Diluent (g)	Filler (g)	Promoter (g)	Curing Agent (g)	Catalyst (g)	Lap Shear Strength (MPa)	Impact Peel Strength (N/Mm)
1	41	23	25	0	5.5	2.2	4.8	0.45	36	34
2	41	23	0	25	5.5	2.2	4.8	0.45	32	-
3	41	17.6	28	0	5.5	2.2	4.8	0.45	35	35
4	41	17.6	0	28	5.5	2.2	4.8	0.5	36	27
5	38	20	32	0	7.2	0.3	5	0.35	35	41
6	38.7	20	23.9	2.7	7.2	0.3	5	0.35	36	45
7	38.7	20	23.5	2.7	7.2	0.3	5	0.35	37	44
8	40	20	21.6	6	7.2	0.3	5.2	0.35	38	32
9	39.5	20	21.6	6.5	7.2	0.3	5.2	0.35	37	28
10	38.7	20	23.9	2.7	9.5	0.3	5.2	0.35	36	34
11	38.7	20	23.9	2.7	8.5	0.3	5.2	0.35	37	34
12	38.7	20	23.9	2.7	8.5	2.2	5.5	0.35	38	27
13	40	18.5	21	6.2	7.2	0.3	5.2	0.35	37	51.1
14	40	17.6	22	6.2	7.2	0.3	4.8	0.35	36	48
15	38	20	22	6.2	7.2	0.3	4.8	0.35	36	39
16	38	20	23.9	2.7	7.2	0.3	5	0.35	36	41
17	38.7	20	23.5	2.7	7.2	0.3	4.8	0.35	37	37
18	32.5	26	21.6	5.3	7.2	0.3	5.15	0.35	37	30
19	38.5	19	21.6	6.2	7.2	0.3	5.05	0.35	37	38
20	38.5	22	23.9	2.7	8.5	0.3	5.2	0.35	38	43
21	27.4	30	28.1	0	7.2	0.3	5.2	0.35	36	40
22	27.4	30	25.5	2.7	7.2	2.2	5.2	0.35	37	27
23	27.4	30	21	6.2	7.2	0.3	5.2	0.35	37	45
24	27.4	30	21	6.2	7.2	0.3	4.8	0.35	36	44.1
25	30.4	30	21	2.7	7.2	0.3	5.2	0.35	37	29
26	27.4	30	23.9	5.3	7.2	0.3	5.2	0.35	37	43
27	30.4	30	26.5	0	7.2	0.3	5.2	0.35	38	42.8
28	30.4	30	26.5	0	7.2	0.3	4.8	0.35	37	38
29	30.4	30	26.5	0	7.2	0.3	5.05	0.35	37	39.7
30	27.4	25	26	6.2	7.2	0.3	5.2	0.35	37	41.8
31	27.4	25	27.1	5.3	7.2	0.3	5.2	0.35	37	43.2
32	27.4	25	27.1	5.3	7.2	2.2	4.8	0.35	37	39.8
33	27.4	25	27.1	5.3	7.2	0.3	5.05	0.35	37	42.1
34	27.4	25	24.5	7.5	7.2	0.3	5.2	0.35	37	38.8
35	27.4	25	24.5	7.5	7.2	0.3	4.8	0.35	37	40.2
36	27.4	25	24.5	7.5	7.2	0.3	5.05	0.35	37	40.7
37	20	30	26.5	8	7.2	0.3	5.2	0.35	35	40.2
38	20	30	26.5	8	7.2	0.3	4.8	0.35	36	38.4
39	20	30	26.5	8	7.2	0.3	5.05	0.35	36	39.8
40	20	25	28.2	6.2	7.2	0.3	5.2	0.35	37	44.5
41	20	25	29.1	5.3	7.2	0.3	5.2	0.35	38	47.2
42	20	25	29.1	5.3	7.2	2.2	4.8	0.35	37	42.1
43	20	25	29.1	5.3	7.2	0.3	5.05	0.35	37	42.8
44	40	15	16.5	8	7.2	0.3	5.2	0.35	38	38.7
45	40	15	16.5	8	7.2	0.3	4.8	0.35	36	34.1
46	40	15	21.8	2.7	7.2	0.3	5.2	0.35	37	41.7
47	40	15	21.8	2.7	7.2	0.3	5.05	0.35	37	40.1
48	40	10	26.8	2.7	7.2	0.3	5.2	0.35	38	43.1
49	40	10	29.1	5.3	7.2	0.3	5.2	0.35	37	39.6
50	40	10	30.1	6.2	7.2	0.3	5.2	0.35	37	39.2
								**Average**	36.68	39.08
							**Standard deviation**		1.02	5.59

**Table 2:** The root mean squared error (RMSE) and normalized squared error (NSE) of the training set and test set for predicting the lap shear strength and impact peel strength using the ANN model.

	Lap Shear Strength		Impact Peel Strength	
	Training Set	Test Set	Training Set	Test Set
RMSE	0.053	0.590	1.730	8.218
NSE	0.002	0.954	0.040	1.452

## Data Availability

Data is contained within the article.
